# Pattern of injuries due to wild animal attack among patients presenting to the emergency department: A retrospective observational study

**DOI:** 10.1016/j.cjtee.2021.09.004

**Published:** 2021-09-20

**Authors:** Pradeep Kumar Singh, S Manwar Ali, Rakesh Vadakkethil Radhakrishnan, Chitta Ranjan Mohanty, Manas Ranjan Sahu, Bishnu Prasad Patro, Ijas MS, Susant Kumar Panda

**Affiliations:** a Department of General Surgery, All India Institute of Medical Sciences, Bhubaneswar, 751019, India; b College of Nursing, All India Institute of Medical Sciences, Bhubaneswar, 751019, India; c Department of Trauma and Emergency, All India Institute of Medical Sciences, Bhubaneswar, 751019, India; d Department of Forensic Medicine and Toxicology, All India Institute of Medical Sciences, Bhubaneswar, 751019, India; e Department of Orthopedics, All India Institute of Medical Sciences, Bhubaneswar, 751019, India; f Department of General Surgery, District Head Quarter Hospital, Angul, Odisha, 759122, India

**Keywords:** Wild animal, Injury severity score, Elephant, Wild boar, Polytrauma

## Abstract

**Purpose:**

The human-wildlife conflicts (HWCs) causing nuisances and injuries are becoming a growing public health concern over recent years worldwide. We aimed to study the demographic profile, mode of injury, pattern of injury, and outcome of wild animal attack victims presented to the emergency department.

**Methods:**

This retrospective cross-sectional study was conducted in the emergency department of a tertiary-care hospital in Eastern India. Data were retrieved from the medical records from May 2017 to May 2021. Patients of all ages and genders attacked by wild animals and secondary injuries were included in this study. Patients with incomplete data, injuries due to the attack of stray and domestic animals and trauma due to other causes were excluded. Demographic profile, mode of injury, the pattern of injury, injury severity score (ISS), radiological pattern, and outcome were recorded. Statistical analysis with R (version 3.6.1.) was conducted.

**Results:**

A total of 411 wild animal attack victims were studied, of which 374 (90.9%) were snakebite injuries and 37 (9.1%) were wild mammalian (WM) attack injuries. The mean age of WM attack victims was 46 years, and the male-to-female ratio was 4:1. Elephant attack injury (40.5%) was the most common WM attack injury reported. Most WM attacks (43.2%) occurred between 4:00 a.m. to 8:00 a.m. The median ISS was 18.5 (13–28), where 54.2% of patients had polytrauma (ISS>15). Elephant attack was associated with a higher ISS, but the difference was not significant compared to other animal types (*p* = 0.2). Blunt trauma was common pattern of injury in the elephant attack injury cases. Lacerations and soft tissue injuries were common patterns in other animal attacks. Among snakebites, neurotoxic was the most common type (55.4%), and lower extremity was the most common site involved.

**Conclusion:**

The young male population is the major victim of HWCs; and elephant is the most common animal involved. There is a need to design scientifically sound preventive strategies for HWCs and to strengthen the preparedness in health establishments to manage victims effectively.

## Introduction

The human-wildlife conflicts (HWCs) causing nuisances and injuries are growing public health concerns over recent years worldwide, especially in the southeast Asian region.^1^^,^^2^ The steadily rising trend in wild animal inflicted injuries substantially contributes to society's overall burden. Notably, the nuisance and injuries among people often give rise to hostility and anger against the involved wildlife, putting challenges for wildlife conservation.^3^ Despite their rarity, wild animal injuries often assume severe forms, resulting in high mortality and morbidity.^4^ Deforestation, urbanization, industrialization, migration, and human intrusion to wild life are some of the prevailing states of affairs that render wild animals losing their natural habitats and increasing their presence over the indwelling human areas.^5^ Earlier literature from India had reported injuries resulting from HWCs with elephants, bear maul, tigers, leopards, hyena, wild boar, and monkeys from some regions, especially from the northern, central, and eastern states.^6^, ^7^, ^8^ Odisha is a tribal-dominated state located on the east coast of India. It has a thick coverage of forest area constituting 37.34% of total land area with diverse flora and fauna.^9^ This makes the inhabitants of this aera more susceptible to injury by wild animals. The victims of wildlife attacks present with varying injury patterns ranging from simple soft tissue injuries to grievous organ injuries and even death.

Hence, the current study aimed at describing the pattern of injuries, mode of injury, and outcome among victims of wild animal attacks presenting to the emergency department (ED).

## Methods

This retrospective observational study was performed in the ED of a tertiary care hospital in eastern India. The approval was obtained from the institute ethics research committee (IEC Ref No: T/IM-NF/Gen.Surg/20/122). The article adheres to the Strengthening the Reporting of Observational Studies in Epidemiology Statement: guidelines for reporting observational studies. All cases of wild animal attacks attending the ED of our institute from May 1, 2017 to May 1, 2021 were included in the study. The inclusion criteria were victims of wild animal attacks regardless of age and gender. The exclusion criteria were (1) incomplete data; (2) injuries due to the attack of stray and domestic animals; and (3) other causes of trauma.

The paper-based records of patients were retrieved from the medical record department, and the data were compiled on a pre-designed structured pro-format and Microsoft Excel worksheet. Data pertaining to the basic demographic details (age and sex), time and place of the animal attack, type of animals, and details of patient occupation were recorded. The mechanism of injury was categorized as direct or indirect. The anatomical site of injury was classified and described as head injury, maxillofacial injury, chest injury, abdominal injury, extremity injury, and soft tissue injury. The pattern of injury was categorized as blunt trauma injury, lacerations, or soft tissue injuries. The severity of the injury of wild mammalians (WM) attacks was calculated using the injury severity score (ISS). The standard assessment and resuscitation protocol for trauma patients was employed to all animal attack victims. All patients having WM bites were treated with anti-rabies vaccination and anti-tetanus injection, while with anti-tetanus injection alone in case of snakebite. The routine investigations like complete blood count, liver function test, renal function test, serum electrolytes, coagulation profile (prothrombin time, activated partial thromboplastin time, international normalized ratio), and clot retraction test were done in all snakebite cases. The emergency procedures perfomed in the ED were recorded. The patient management approach was categorized as conservative or surgical. The outcome was recorded as admitted, discharged, or referred.

Statistical analysis was done by R version 3.6.1. Categorical variables were expressed as frequency and percentage. The Shapiro-Wilk test determined the normalcy of numerical data. Numerical parametric variables were expressed as mean ± standard deviation and non-parametric median with interquartile range (IQR). Kruskal-Wallis test was used to compare the ISS between different groups of animals. A value of *p* < 0.05 was regarded as statistically significant.

## Results

**Fig. 1:**
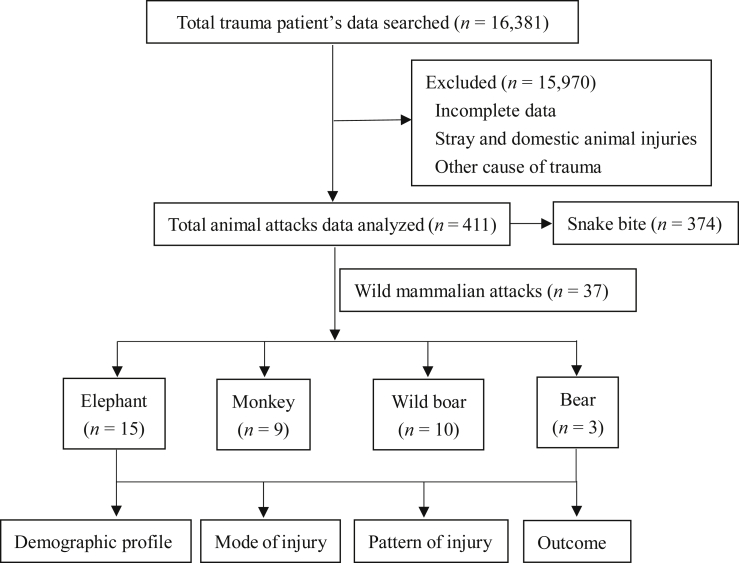
Flow diagram of the study.

**Fig. 2:**
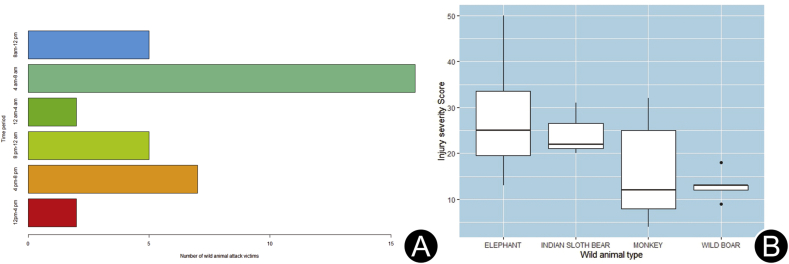
(A) Bar plot showing distribution of time of wild animal attack; (B) Boxplot showing comparison of injury severity score between different types of animal attack victims.

**Fig. 3:**
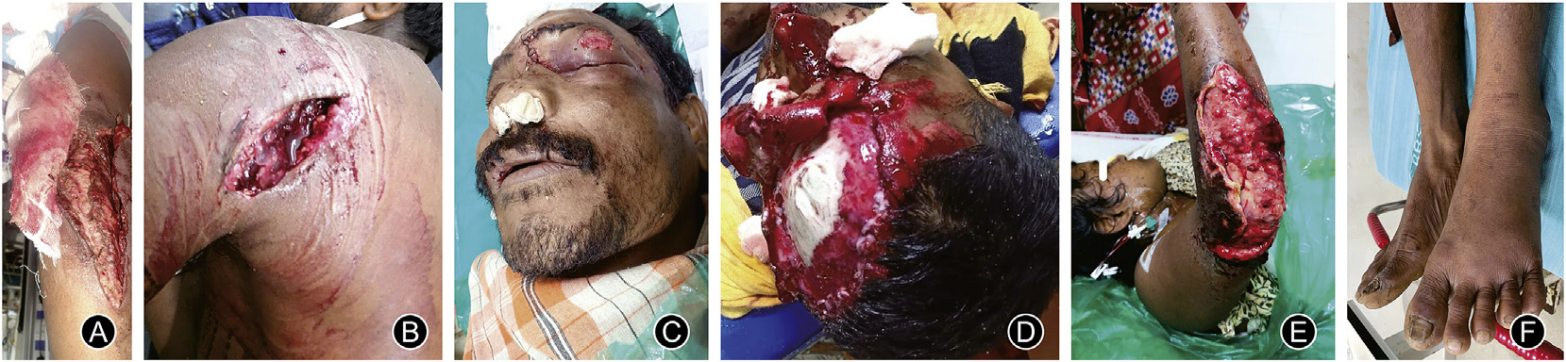
Injuries due to animal attack. (A) Victim of elephant attack (laceration of forearm); (B) Victim of bear attack (laceration of trapezius region); (C) Victim of wild boar attack (maxilla-facial injury); (D) Victim of monkey attack (injury of scalp due to RTA); (E) Victim of elephant attack (wound after debridement); (F) Victim of vasculo-toxic snake bite (swelling of lower extremity).

**Fig. 4:**
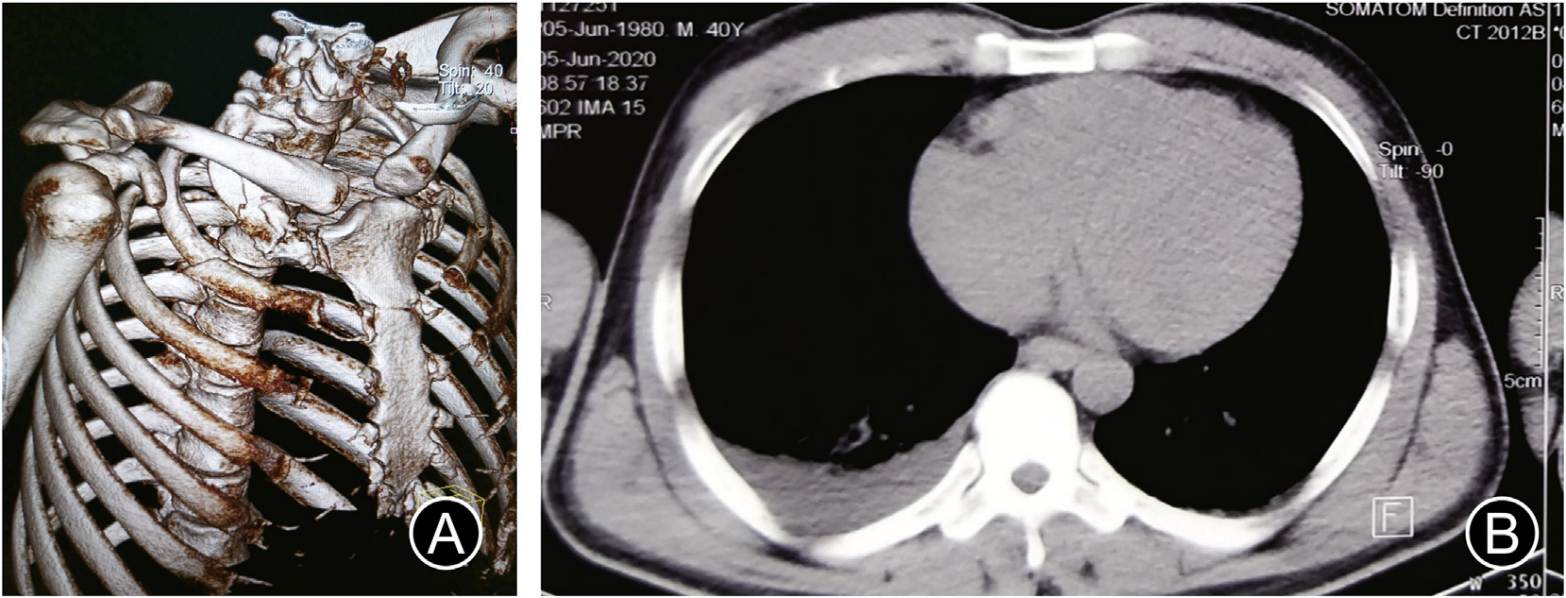
Radiological findings in elephant attack: (A) 3D reconstruction of CT chest showing 1st and 2nd rib-fracture; (B) Cross-sectional image of CT thorax showing haemothorax.

The flow diagram of the study is shown in Fig. 1. A total of 37 WM attack victims and 374 snakebite cases were included for analysis. Snakebite (90.9%) was the most reported wild animal attack. Among WM attacks, elephant attack (40.5%) was the most commonly reported. The year-wise cases of wild animal attack are shown in Table 1. Amongst the snakebite victims, the majority had neurotoxic envenomation (55.4%), with lower extremities being the most common bitten site (72.9%). The mean age of WM attack victims was (46 ± 15.2) years, and the male-to-female ratio was 4:1. The mean age of snakebite victims was (36 ± 14.3) years, and the male to female ratio of snakebite cases was 3:1. Approximately, 43.3% of snakebite cases occurred in residential areas. Amongst the location of WM attacks, most attacks (40.4%) occurred in rural residential areas, followed by urban residential areas (35.2%), where 25.5% occurred in farmland and forest. The major mode of injury was a direct attack by the WMs. Indirect mode of injury (road traffic accidents and fall from height) accounted for 66.6%, 43.2%, and 12.8% in the case of monkeys, wild boar, and elephant, respectively. All attacks by wild animals were unprovoked, except one provoked attack by a monkey and another by a bear. The early morning time (4:00 a.m. to 8:00 a.m.) was the most common time of attack by wild animals (43.2% of cases), and details of injury times are depicted in Fig. 2A. The most common time of snakebite (43%) was 12:00 p.m. to 4:00 p.m. The pattern of injuries due to WM attacks and the radiological findings are detailed in Table 2. Fig. 3 (A to E) shows the injuries due to different types of wild animal attacks. The radiological findings in elephant attacks are shown in Fig. 4. Lacerated wounds and soft tissue injuries were commonly reported in the bear, wild boar, and monkey attacks.

**Table 1:** Year wise cases of wild mammalian attacks and snake bites (*n*).

Year	Elephant attack	Wild boar attack	Monkey attack	Bear attack	Snake bite	Total
2017	1	3	0	0	75	78
2018	1	1	2	2	121	127
2019	1	0	4	0	144	159
2020	6	3	1	1	101	112
2021(till May)	6	3	2	0	34	45

**Table 2:** The pattern of injuries due to wild animal attacks and the radiological findings. (*n* = 37).

Animal type	Injury pattern	Radiological findings		
		FAST	X-ray	CT scan
Elephant (*n* = 15)	Head injury (*n* = 4) Blunt trauma chest (*n* = 11) Blunt trauma abdomen (*n* = 8) Penetrating chest injury^a^ (*n* = 1) Extremity injuries (*n* = 10)	Positive (*n* = 4)	B/L haemothorax with rib fracture (*n* = 2)	Head CT: intra-ventricular haemorrhage with pneumocephalus (*n* = 1) Head CT: WNL (*n* = 3) Chest CT: B/L haemothorax with rib^b^ (*n* = 2) Right haemothorax (*n* = 2) Right pneumothorax (*n* = 2) T12 spine compression^b^ (*n* = 1)
Monkey (*n* = 9)	Head, maxillofacial and extremity injuries^a^ (*n* = 5) Distal tibia and lumbar spine fracture^a^ (*n* = 2) Abrasions, lacerations, and soft tissue injury^a^ (*n* = 2)	Negative^a^ (*n* = 6)	Fracture distal tibia^a^ (*n* = 1) Fracture distal tibia and talus^a^ (*n* = 1)	3D face reconstruction: right mandible, condyle, and para symphysis^a^^b^ (*n* = 1) Displaced^b^ of dento-alveolar complex (*n* = 1) L1 spine body^a^^b^ (*n* = 1) L2-L3 spine compression^a^^b^ (*n* = 1)
Wild boar (*n* = 10)	Laceration of upper extremity (*n* = 3) Laceration of lower extremity (*n* = 4) Soft tissues injuries (*n* = 4) Head and maxillofacial injuries^a^ (*n* = 2) Extremity injuries^a^ (*n* = 2)	NA	Left distal radius^a^^b^ (*n* = 1) Fifth metatarsal^a^^b^ (*n* = 1)	3D face reconstruction: nasal bone^a^^b^ (*n* = 1)
Bear (*n* = 3)	Head injury (*n* = 1) Maxillofacial injuries (*n* = 2) Soft tissue injuries (*n* = 2) Laceration of upper and lower extremity (*n* = 3)	NA	Compound fracture of tibia (*n* = 1)	3D face reconstruction: zygomatic bone^b^ (*n* = 1)

FAST: focused assessment with sonography in trauma; NA: not advised; WNL: within normal limits.

a Indirect injury.

b Fracture.

The median ISS among the victims was 18.5 (13–28), where 54.2% had polytrauma (ISS>15). A higher ISS was observed in victims of elephant attacks in comparison to others, but the comparison between all animal types was not significant (*p* = 0.2). The ISS across various wild animal inflicted injuries is depicted in the boxplot (Fig. 2B). In the ED, 19 patients (51.3%) received wound suturing and dressing, 11 patients (29.7%) required splint or cast application. K-wire fixation was done in 2 patients in the ED. The chest tube insertion was required in 6 (16%) patients, out of which 2 patients had bilateral chest tube insertion. Of the total WM attack patients, 16 (43.2%) were discharged after primary treatment from the ED; 6 patients (16.2%) were admitted to intensive care unit (ICU); 14 patients (37.8%) were admitted to the ward, out of which 7 patients underwent orthopedic surgical procedures; 2 patients underwent neurosurgical procedures; and 1 (2.7%) patient was referred. One patient following an elephant attack died during treatment in ICU. Totally 359 (95.9%) snake bite patients received anti-snake venom medication in the ED. Two-hundred and thirty-five (62.8%) patients were discharged after treatment in the ED, 128 (31.1%) were admitted in the ward, and 11 (3.2%) admitted in ICU.

## Discussion

In the ED, wild animal attack injuries presented as soft tissue injury, lacerations of different body parts, and blunt trauma to the head, chest, abdomen, and extremities, similar to injuries due to road traffic accidents and physical assault. The indirect injuries due to wild animal attack that resulted in road traffic accidents or fall from height is also similar to conventional injuries. The wild animals can inflict serious tissue injury with their powerful jaws and grinding teeth.^10^ Unlike conventional wounds due to other causes of trauma, the risk of wound infection is significant, and systemic illnesses, such as brucellosis, leptospirosis, or tularemia, can follow the injury.^10^

The present study described the socio-demographic profile of victims of wild animal attacks, their mode, and pattern of injury. The young, active male population were the major victims of wild animal attacks, and most reported were snakebites and elephant attacks. The victims suffered mostly blunt trauma in case of an elephant and bear attack. Soft tissue injuries and lacerations were the predominant injury pattern in the bear, wild boar, and monkey attacks. The incidence of HWCs is taking a steady trend over recent years globally, especially in the southeast Asian region. Eastern India is geographically and topographically different from the rest of the nation, with dense forest coverage accommodating a rich diversity of flora and fauna.^9^ Being a tribal-dominated state, Odisha marks the presence of human indwelling areas near this large chunk of wildlife, making inhabitants of this part of the country most susceptible to attacks from this wildlife.

In the present study, snakebites accounted for most of the wild animal attacks (90%), with neurotoxic bites outnumbered vasculotoxic bites (55% *vs.* 45%). The findings are in contrast with earlier studies from India that reported Russel's viper is the most common snake species in the country known to produce vasculotoxic envenomation.^11^^,^^12^ However, one previous study from India by Vora et al.^13^ also reported more of neurotoxic envenomation in their series of snakebite victims. The eastern India region is notorious for harboring sizable share of elapid snake species like Indian cobra, and commonly krait produces neurotoxic envenomation. Furthermore, the majority of the bites occurred in the lower extremities (73%), with peak incidents during the rainy seasons (June to September), which agrees with findings of Bhuiyan et al.^14^ and Suraweera et al.^12^

In our study, males were the predominant victims (80%) of WM attacks, which agrees with the findings of other studies.^7^^,^^8^^,^^15^^,^^16^ It is because males are more involved in outdoor activity than females. The mean age of patients was 46 years, which is similar to earlier literature that highlighted a higher incidence of animal attack injuries in the third decade of life.^7^^,^^15^ Most of our patients (40%) were from rural areas, which agrees with the study by Gilyoma et al.,^15^ in which most patients belong to rural areas. Conversely, Bombieri et al.^16^ reported the prevalence of wild animal attacks on humans in urban dwellers in their study based in the North American region. In our study, the majority (44%) of the patients sustained WM attacks during early morning hours, and most of the attacks are unprovoked. The findings are in line with reports of Bhat et al.,^7^ the wild animals invade farmlands and nearby village areas to consume crops and drinking water from ponds during night or morning time and may encounter people who were on their way to work during these morning hours. In the current study, most (40%) of the WM attack occurred in rural residential areas and 35% got attacked in urban residential areas. Only 25% occurred in farmland or forest areas, signifying wider intrusion of wildlife into the human indwelling areas. This contrasts with earlier studies reported from other locations of our country, which reported most attacks occurred in forest and adjacent farmlands than residential areas.^7^^,^^8^ Our centre is in the capital city of Odisha state, having proximity to the nearby dense forest areas, and likely to get patients who suffered injuries from nearby localities. There were many reports of wild animal attacks in Odisha's rural and urban residential areas during recent times.^17^, ^18^, ^19^, ^20^ Furthermore, the study centre is a tertiary care facility and received referral cases from rural, semi-urban, or urban localities. Most of those patients with minor injuries that had occurred in forests or farmlands would have received treatment from nearby local health facilities. Earlier studies also highlighted that most animals inflicted injuries result in mild trauma and can be managed with ambulatory treatment without requiring further hospitalization.^10^^,^^15^

Most elephant attacks were reported in the early morning hours as people went for their morning work. The victims of elephant attacks had a higher ISS of 32 compared to other WMs in our study, suggesting fatal injuries.^21^ The findings agree with Acharya et al.,^2^ who reported attacks by elephants had a higher odd of being killed in comparison to leopard, tiger, and rhinoceros in a study conducted in our neighbouring country of Nepal. The pattern of injury inflicted by elephants to humans is quite different due to the disparity in their sizes. They commonly use blunt force to attack, while penetrating injuries by tusk is uncommon. Blunt chest and abdomen trauma were the most common reported pattern of injury, though head and maxilla-facial injury also reported which is similar to other studies.^2^^,^^22^ Nearly half of our patients suffered an attack by elephants. The menace of wild elephants is taking an alarming toll in the state, where a recent report revealing nearly 680 peoples were killed by elephants attack alone over the last 8 years.^23^ Considering the rising trend of wild animal attack in the state, the government initiated several efforts to safeguard both human and wildlife but are suboptimal to cater the actual need. The state government launched monetary compensation for victims of wildlife attacks decades ago and has been periodically revised over the years, especially for fatal attacks, which is currently running at a sum of 6800 USD. To rescue the elephants in distress, the forest department is also equipped with special vehicles to drain them back to their natural habitats.^23^

Monkey**-**related injuries were mostly indirect in our study, as the victims suffered from road traffic accidents or fall from height after the attack by the monkeys. Those sustained direct injuries were soft tissue injuries. The monkey-inflicted injuries are also on the rise in the state over recent periods.^18^^,^^24^, ^25^, ^26^ The wild boars are plant-eating herbivorous animals which accounts for injuries in 27% of WM attack victims. Wild boar attack injuries mostly took place in villages where they enter for food and attack the locals or the farmers while working in the paddy field. They mostly entered the crop field during the night hours to eat crop. Most victims suffer laceration of the lower and upper extremities. Increased incidence of wild boar attacks also being reported from urban areas in recent times, where they typically attack multiple victims at a time and mostly following cluster attacks.^17^^,^^19^ Bear is the most common wild animal involved in HWCs in India,^7^^,^^8^^,^^27^ though it is contrary to the results of our study. The victims commonly sustain tearing, crushing, or penetrating injuries as the animal attack with powerful paws, claws, and teeth. The extremity injuries, maxilla-facial injuries, chest injuries, and soft tissue injuries are likely encountered and can be grievous with even fracture of extremities. The case fatality rate is also high. The attacks are implicated as a measure of self-defense or during provocation.^7^^,^^27^

In the current study, injury patterns in most of the patients were lacerations and soft tissue injuries in the upper and lower extremities, except in victims of elephant attacks where blunt injuries to the chest and abdomen predominated along with extremity injuries. The findings are consistent with earlier studies that reported a predominance of soft tissue injuries and extremity injuries among animal attack victims.^15^^,^^28^ Some authors have also highlighted the reason behind these extremity injuries as the animals are at ease to injure the moving body parts.^7^^,^^15^^,^^28^ In our study, 54% of the patients underwent suturing and dressing in the ED. Around 31% of patients required splint or cast application due to fracture or dislocation of the extremities. This is due to the injury pattern inflicted by wild animals by means of scratches and punctures with sharp teeth and nails and blunt force in large mammals. The injuries sustained from animal attacks range from minor bruises and contusions to deeper extensive injuries such as puncture wounds, avulsions, separation of punctured flaps, and even amputations.^29^^,^^30^ Our findings of suturing and dressing in most victims are consistent with the study of Gilyoma et al.^15^ that reported surgical wound debridement with either primary or delayed closure was the most common procedure done on their patients of the animal attack.

The complications from animal bites are mechanical injury by bite itself, local bacterial infection, and systemic infection. In animal bites, effective wound management and prevention of infection should be the key goals. Meticulous examination to determine the extent of tissue damage, with attention for penetration into joint spaces and tendon sheath, and cleaning of the wound, including aggressive irrigation and debridement of devitalized tissue, is essential.^10^ The maxillofacial, perineal, and hand injuries can be challenging due to the proximity of anatomically important structures and should be managed effectively by plastic surgeons. Imaging should be done to detect fractures and rule out retained foreign bodies such as animal teeth.^10^ Prophylactic broad-spectrum antibiotics to prevent and treat infections, along with anti-rabies treatment, is essential.^10^

The current study has some limitations which need to be acknowledged while interpreting the study results. It was a single-center study, limiting the generalizability of study findings. Secondly, as our center is a tertiary care hospital, we received only a few referred cases. As most of the cases were treated in local hospitals or referred to state medical colleges, fewer patients were presented to ours. However, the actual magnitude of incidents is relatively high.

The study shed light on the key epidemiological parameters, including the mode and injury patterns among victims of wild animal attacks from a state having diverse flora and fauna. The young male population were the major victims of wild-animal attacks, with the most common animal involved being the elephant. Most victims had polytrauma, and the pattern of injury mostly specific for each animal. Future multicentre prospective studies are warranted to capture the exact magnitude of HWCs in this part of the world.

## Funding

Nil.

## Ethical statement

The approval was obtained from the institute ethics research committee (IEC Ref No: T/IM-NF/Gen.Surg/20/122).

## Declaration of competing interest

The authors declare no conflicts of interest.
